# Dataset from HDX-MS Studies of IgG1 Glycoforms and Their Interactions with the FcγRIa (CD64) Receptor

**DOI:** 10.6028/jres.126.010

**Published:** 2021-06-17

**Authors:** Kyle W. Anderson, Kerry Scott, Ioannis L. Karageorgos, Elyssia S. Gallagher, Venkata S. Tayi, Michael Butler, Jeffrey W. Hudgens

**Affiliations:** 1 National Institute of Standards and Technology, Gaithersburg, MD 20899, USA; 2 Institute for Bioscience and Biotechnology Research, 9600 Gudelsky Drive, Rockville, MD 20850, USA; 3 Department of Microbiology, University of Manitoba, Winnipeg, MB R3T 2N2, Canada; 4 National Institute for Bioprocessing Research and Training, Foster Avenue, Mount Merrion, Blackrock, Co. Dublin, Ireland

**Keywords:** antibody-receptor interaction, chromatography, glycosylation, hydrogen-deuterium exchange, mass spectrometry, monoclonal antibody, peptide, precision, protein, proteolysis, proteomics, receptor

**Data DOI:** https://doi.org/10.18434/mds2-2365

## 1 Summary

Monoclonal antibody (mAb) pharmaceuticals account for the emergence of safer, targeted therapeutics currently addressing cancer [1], autoimmune conditions [2-4], osteoporosis, [5] macular degeneration [6], migraines [7], and infectious diseases including SARS-CoV-2 [8, 9]. Manufacture of the ≈ 100 approved mAb biopharmaceuticals, produced from cultured mammalian cells, amounts to tens of metric tons of material annually [10]. A feature common to mAb biotherapeutics is the attachment of glycans at asparagine 297 (N297) in the Fc domain, which affects antibody conformation and conformational dynamics. Changes in these dynamical properties can affect binding with receptors. Thus, the glycan distribution is a critical quality attribute that is carefully monitored during mAb manufacture [11-13]. HDX-MS studies have proved important for characterizing the dynamics of IgG1 glycoforms [13-20] and their interactions with receptors [15, 16]. Moreover, measurements of the differences in molecular dynamics of mAb glycoforms can provide information useful for evaluating similarities between an innovator biotherapeutic and a candidate biosimilar.

**Fig. 1:**
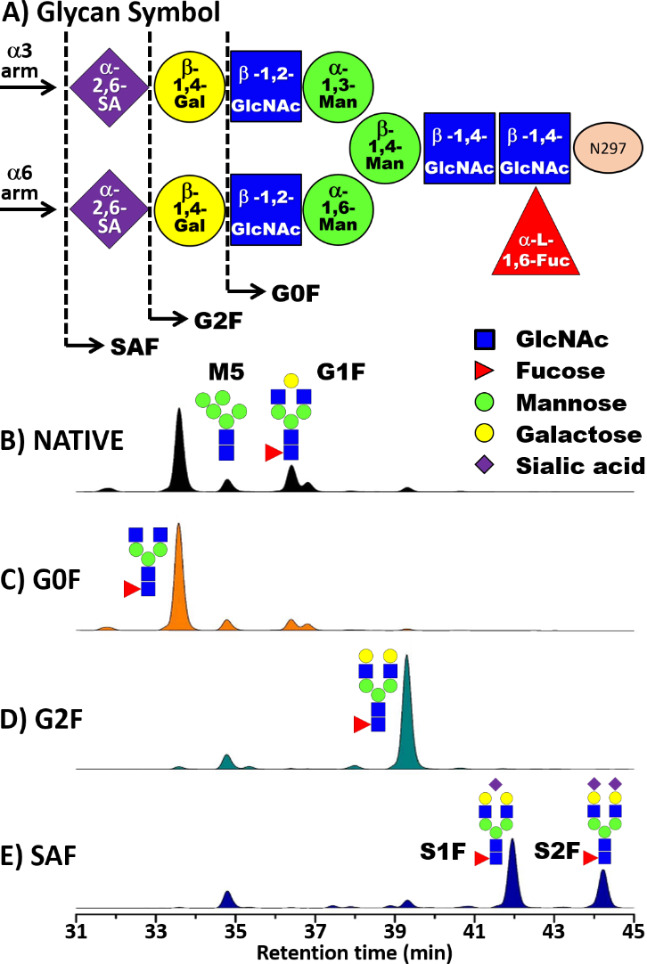
IgG1 glycoforms of aIL8hFc [21] and hydrophilic interaction liquid chromatography (HILIC) traces of glycans released from IgG1 materials by peptide-N-glycosidase F and labeled with fluorescent 2-aminobenzamide [22]. A) Symbolic representation of glycan structure in aIL8hFc with bonding denoted within each symbol. An arrow and dashed line demark the composition for each subject glycoform. B) HILIC trace of native aIL8hFc, C) HILIC trace of the G0F sample, D) HILIC trace of the G2F sample, and D) HILIC trace of the SAF sample.

This document presents hydrogen-deuterium exchange mass spectrometry (HDX-MS) data from measurements of three purified IgG1 glycoform samples (Fig. 1), predominantly G0F, G2F, and SAF, in isolation and in complexation with the high-affinity receptor, FcγRIa (CD64). The IgG1 antibody used in this study, aIL8hFc, is a murine-human chimeric IgG1, which inhibits IL-8 binding to human neutrophils [23].

### 2 Data Specifications

**Table:** 

**NIST Operating Unit(s)**	Materials Measurement Laboratory, Biomolecular Measurement Division
**Format**	CSV, PDF
**Instrument**	Thermo LTQ Orbitrap Elite mass spectrometer (Thermo Fisher, San Jose, CA).
**Spatial or Temporal Elements**	N/A
**Data Dictionary**	N/A
**Accessibility**	All datasets submitted to *Journal of Research of NIST* are publicly available.
**License**	https://www.nist.gov/director/licensing

## 3 Methods

### 3.1 Reagents and Materials used for HDX-MS measurements^1^1 Certain commercial equipment, instruments, or materials are identified in this paper to foster understanding. Such identification does not imply recommendation or endorsement by the National Institute of Standards and Technology, nor does it imply that the materials or equipment identified are necessarily the best available for the purpose.

All chemicals were purchased from Sigma-Aldrich (St. Louis, MO, USA) unless otherwise noted. D_2_O (99.96 mole % D) was obtained from Cambridge Isotope Laboratories Inc. (Andover, MA, USA). Tris(2-carboxyethyl)phosphine hydrochloride (TCEP) and guanidine hydrochloride (GdmHCl) were purchased from Thermo Scientific (Rockford, IL, USA).

Soluble FcγRIa/CD64A receptor of UniProt accession number P12314 (> 90% purity determined by SDS-PAGE) expressed from HEK293 cells and lyophilized from sterile, pH 7.4, phosphate-buffered saline (PBS), was acquired from Sino Biological (Catalog# 10256-H08H, Beijing, China).

aIL8hFc mAbs were expressed from CHO DP-12 clone#1934 cell line (American Type Culture Collection, Manassas, VA, USA; Catalogue # CRL-12445). Briefly, cells were inoculated at 2.5x10^5^ cells/mL into 250 mL shake flasks each containing 80 mL Biogro CHO media (Biogro Technologies Inc, Winnipeg, MB, Canada) with 25 mol/L glucose and 0.5g/L yeast extract (BD Diagnostics, Sparks Glencoe, MD, USA). Cells were cultured by incubating the shake flasks in a humidified incubator (Nuaire, Plymouth, MN, USA) at 120 rpm, 10% CO_2_ and 37 ^o^C. After 4 days growth, the cultures were centrifuged at 1500 *g*_n_ for 5 min to collect the culture supernatant that was filtered through a 0.2 μm Steritop filter (EMD Millipore, Etobicoke, ON, Canada).

IgG1 glycoforms of aIL8hFc were prepared by solid-phase enzymatic remodeling [24]. Briefly, mAbs from culture supernatant were bound to a Protein-A HP SpinTrap affinity column (GE Healthcare, Fairfield, CT, USA) using conditions typical for mAb purification. After washing out non-bound impurities by a neutral pH buffer (phosphate buffer saline), antibodies were subjected to enzymatic modification directed to a targeted glycan profile [24]. The antibodies were then eluted with a low pH buffer (0.1 mol/L Glycine-HCl, pH 2.7) and then neutralized to pH 7.2 with Tris-HCl buffer (pH 9.0). The glycoform populations were determined by releasing N-glycans from IgG1 with peptide-N-glycosidase F; linking filtered, released glycans with fluorescent, 2-aminobenzamide (2AB) label; separation of tagged glycans with hydrophilic interaction liquid chromatography (HILIC), and evaluation of glycan abundance from peak areas of observed fluorescent signal [22].

### 3.2 Composition of FcγRIa and aIL8hFc

Soluble FcγRIa/CD64A receptor of UniProt accession number P12314 (> 90% purity) expressed from HEK293 cells and lyophilized from sterile, pH 7.4, phosphate-buffered saline (PBS), was acquired from Sino Biological (Catalog# 10256-H08H) Beijing, China). Soluble FcγRIa receptor comprises 284 amino acids containing D1, D2 and D3 of the ectodomain. The sequence of soluble FcγRIa between residues 16 and 282 shares the sequence observed in the crystal structure of FcγRIa (Protein Data Bank identifier (PDB): 3RJD) [25]. The sequence of soluble FcγRIa between residues 21 and 282 shares the same sequence as observed in the crystal structure of the FcγRIa—Fc complex (PDB: 4ZNE) [26].

The glycan distribution of the FcγRIa material used in these experiments was not measured. Previous studies have found that soluble FcγRIa receptor contains six asparagine sites occupied by 30 different glycosylation structures, comprising ≈ 18% of total molecular weight. The N-glycan distribution comprises complex (70%) high mannose (9%), and hybrid (3%) structures. Most glycans contain core fucosylation (67%) and a small portion (12%) are capped with one Neu-5-Ac sialic acid [27].

Each IgG1 glycoform is named by the predominate glycan structure bound to N297, e.g., G0F refers to aIL8hFc-G0F. HILIC traces of released glycans reveal that the aIL8hFc samples labeled G0F, G2F, and SAF contain other glycoforms (Table 1) [22]. All samples contain small fractions of M5, a high mannose glycoform and G1F (Fig. 1). SAF contains some small fractions of S1F glycan chains terminated with one Neu-5-Ac sialic acid. Since S1F and S2F were prepared using the α(2-6) linkage enzyme, human sialyltransferase, both sialylated structures have α(2-6) linkages.

**Table 1:** Glycoform relative abundances (%) within each aIL8hFc variant sample, as determined from integrated fluorescent peak areas of 2AB-tagged glycans separated by HILIC. Measurement uncertainties are 1*s* ≈ 1%.

Sample	G0F, %	M5, %	G1F, %	G2F, %	S1F, %	S2F, %
G0F	82	7	11			
G2F		7		93		
SAF		11		4	54	31

The study materials were examined for post translational modifications by tandem mass spectrometry (MS/MS). MS/MS measurements of the peptic peptides of each aIL8hFc glycoform detected oxidation only on M252 in peptide ^241^FLFPPKPKDTLM^252^. Integrated MS peak areas of this peptide revealed the degrees of oxidation: G0F (0.8% ± 0.1%), G2F (1.6% ± 0.1%), and SAF (2.2% ± 0.1%), where the uncertainty denotes one sample standard deviation (1*s*). MS/MS data did not detect oxidation in the FcγRIa material. Phosphorylation and deamidation were not detected in aIL8hFc glycoforms or FcγRIa.

Table 2 lists the amino acid sequences for aIL8hFc and soluble FcγRIa (CD64). For the convenience of direct comparisons with other IgG1s including NISTmAb reference material, we apply the EU numbering system to the heavy chain (HC) of aIL8hFc without adjustments for sequence variation. This straightforward numbering extends the heavy chain sequence numbers to a noncanonical −4, which accounts for the slightly longer $V_{H}$ sequence. Although the present numbering scheme is nonstandard, this numbering facilitates direct comparisons of aIL8hFc with many other IgG1s. With this EU numbering scheme aIL8hFc has the same residues and sequence numbers of residues across the $C_{H}1$ (HC 118-215), hinge (HC 216-230) and Fc (HC 231-446) heavy chain regions including correspondence with N297. As expected for comparisons of V_H_ fragments, residues in the $V_{H}$ regions of aIL8hFc differ substantially from other IgG1s. For example, although the light chains (LC) of aIL8hFc and the NISTmAb reference material share the same $C_{L}$ (LC 113-219) sequence, the sequence similarity of their $V_{L}$ (LC 1-112) domains is only 69% [28].

**Table 2:** Amino acid sequences of aIL8hFc and soluble FcγRIa.

**aIL8hFc Heavy Chain:** 1 EVQLVQSGGG LVQPGGSLRL SCAASGYSFS SHYMHWVRQA PGKGLEWVGY IDPSNGETTY 61 NQKFKGRFTL SRDNSKNTAY LQMNSLRAED TAVYYCARGD YRYNGDWFFD VWGQGTLVTV 121 SSASTKGPSV FPLAPSSKST SGGTAALGCL VKDYFPEPVT VSWNSGALTS GVHTFPAVLQ 181 SSGLYSLSSV VTVPSSSLGT QTYICNVNHK PSNTKVDKKV EPKSCDKTHT CPPCPAPELL 241 GGPSVFLFPP KPKDTLMISR TPEVTCVVVD VSHEDPEVKF NWYVDGVEVH NAKTKPREEQ 301 YNSTYRVVSV LTVLHQDWLN GKEYKCKVSN KALPAPIEKT ISKAKGQPRE PQVYTLPPSR 361 EEMTKNQVSL TCLVKGFYPS DIAVEWESNG QPENNYKTTP PVLDSDGSFF LYSKLTVDKS 421 RWQQGNVFSC SVMHEALHNH YTQKSLSLSP GK
**aIL8hFc Light Chain:** 1 DIQMTQSPSS LSASVGDRVT ITCRSSQSLV HGIGETYLHW YQQKPGKAPK LLIYKVSNRF 61 SGVPSRFSGS GSGTDFTLTI SSLQPEDFAT YYCSQSTHVP LTFGQGTKVE IKRTVAAPSV 121 FIFPPSDEQL KSGTASVVCL LNNFYPREAK VQWKVDNALQ SGNSQESVTE QDSKDSTYSL 181 SSTLTLSKAD YEKHKVYACE VTHQGLSSPV TKSFNRGEC
**Soluble FcγRIa:** 3 MWFLTTLLLW VPVDGQVDTT KAVITLQPPW VSVFQEETVT LHCEVLHLPG SSSTQWFLNG 61 TATQTSTPSY RITSASVNDS GEYRCQRGLS GRSDPIQLEI HRGWLLLQVS SRVFTEGEPL 121 ALRCHAWKDK LVYNVLYYRN GKAFKFFHWN SNLTILKTNI SHNGTYHCSG MGKHRYTSAG 181 ISVTVKELFP APVLNASVTS PLLEGNLVTL SCETKLLLQR PGLQLYFSFY MGSKTLRGRN 241 TSSEYQILTA RREDSGLYWC EAATEDGNVL KRSPELELQV LGLQ

### 3.3 Peptide Identifications from Mass Spectrometry Data

Peptic peptides of soluble FcγRIa and aIL8hFc-control were generated by passing 20 pmol of protein through an Enzymate BEH pepsin digestion column (2.1 x 30 mm, 5 μm bead; Waters, Milford, MA, USA) and identified using MS/MS on the Thermo LTQ Orbitrap Elite mass spectrometer. One full mass spectral acquisition triggered six scans of MS/MS with activation by collision-induced dissociation (CID) on the most abundant precursor ions. Peptides were identified by the MASCOT (Matrix Science Inc., Boston, MA, USA) database search engine with the following parameters: enzyme, none; oxidation (M) as a variable modification; MS tolerance, 20 ppm; MS/MS tolerance, 0.6 Da; peptide charge of +2, +3, and +4. Glycopeptides were identified by the Byonic software (Protein Metrics, San Carlos, CA, USA). Byonic searches were performed with the following search parameters: digestion cleavages, C-terminal of residues for pepsin (A, C, E, F, G, L, Q, S, T, V, W); missed cleavages, 6; MS tolerance, 10 ppm; MS/MS tolerance, 0.05 Da; glycan modifications, specific masses to FcγRIa and aIL8hFc-control, two common modifications per peptide, and at most 1 rare modification per peptide.

### 3.4 Bottom-up HDX-MS Measurements

HDX-MS data were collected using the same stock reagents, pH and salt concentrations, and chromatography solutions. Measurements involving the receptor used soluble CD64A/FcγRIa of the same lot number. The HDX-MS data reported herein contain no adjustments for deuterium back-exchange.

To maximize disulfide reduction efficacy, fresh TCEP solutions used in these experiments were prepared daily. This study followed bottom-up HDX-MS methods described elsewhere [29, 30]. For the present HDX-MS analyses, the FcγRIa and aIL8hFc variant protein stocks were diluted in H_2_O buffer (10 mmol/L sodium phosphate, 137 mmol/L sodium chloride, 2.7 mmol/L potassium chloride at pH 7.4) to prepare the following samples: aIL8hFc, aIL8hFc-G0F, aIL8hFc-G2F, and aIL8hFc-SAF at 2 µmol/L final concentration; FcγRIa at 4 µmol/L final concentration; FcγRIa at 4 µmol/L plus each aIL8hFc variant at 2 µmol/L final concentration. HDX-MS experiments used FcγRIa of the same lot number, and all experiments were conducted using the same stock reagent and chromatographic solutions.

All samples were equilibrated at 1 °C. HDX was conducted on an HDX PAL robot (LEAP Technologies, Carrboro, NC, USA). Protein solutions (5 µL) were diluted into 31 µL D_2_O buffer (10 mmol/L sodium phosphate, 137 mmol/L sodium chloride, 2.7 mmol/L potassium chloride at pD 7.4) at 25 °C. After immersion in D_2_O solution for selected times (*t*_HDX_ = (0, 30, 300, 900, 3600, and 14400) s) the HDX sample was quenched by mixing with 30 µL quench buffer (4 mol/L GdmHCl, 0.2 mol/L sodium phosphate, 0.5 mol/L TCEP at pH 2.5) at 1 °C. This solution was injected into a liquid chromatography apparatus that housed its LC connection lines and valves in a refrigerated compartment at ≈ 1 °C. The quenched solution flowed through the immobilized pepsin column for 3 min at 15 °C.

The peptic peptides in the solution digest were trapped on a C18 guard column (1 °C, 1.0 mm dia. x 10 mm length, 5 µm particles; Grace Discovery Sciences, Deerfield, IL, USA) and separated with a C18 analytical column (1 °C, 1.0 mm dia. x 50 mm length, 1.9 µm particles, Hypersil GOLD; Thermo Scientific, Rockford, IL, USA). via a Thermo Scientific Ultimate NCS-3600RS binary pump with a 9.5 min gradient operated with a binary mixture of solvents A and B at 50 µL/min flow rate. The gradient settings used were: 5% to 35% solvent B for 3 min, 35% to 60% solvent B for 5 min, 60% to 100% solvent B for 0.5 min, isocratic flow at 100% solvent B for 0.5 min, and a return in 5% solvent B for 0.5 min. Solvent A was water containing 0.1% formic acid and solvent B was 80% acetonitrile and 20% water containing 0.1% formic acid.

Peptides were measured on a Thermo LTQ Orbitrap Elite mass spectrometer. The instrument settings were: spray voltage, 3.7 kV; sheath gas flow rate, 25 (arbitrary units); capillary temperature, 275 °C. In the Orbitrap stage MS spectra were acquired with the resolution set at 25,000 [31]. HDX-MS experiments performed on each protein sample comprised three measurements of $D_{i}^{peptide}\left(t_{HDX}\right)$ for each peptic peptide. The HDX data reported here contain no adjustments for deuterium back-exchange during the analysis. Fully deuterated samples of Fab fragment of NISTmAb back-exchange observed under like conditions in the present instrumentation ranged from 15% to 30%, depending on sequence [29, 32]. Simulations of the analysis of peptides by integration of back-exchange rate coefficients, derived from public spreadsheets, estimated similar back-exchange effects [33-36].

For each measurement the program, HDX Workbench [37], reports ${\%E}_{i,X}^{peptide}\left(t_{HDX}\right)$, which is the percent of peptide undergoing deuterium exchange, as determined from the mass centroid, obtained for the ith measurement of a peptide in state X (e.g., apo- and holo-glycoform) [31]. Here, $t_{HDX}$ is the interval that the protein resides in a D_2_O solution, and the recovery parameter is set at 100%. Deuterium mass *D* of a peptide from state X is computed using:

$D_{X}^{peptide}\left(t_{HDX}\right)=\frac{{\%E}_{i,X}^{peptide}\left(t_{HDX}\right)F^{D_{2}O}\left(n-p-2\right)\left(m_{D^{+}}{-\;m}_{H^{+}}\right)}{100\%\;}$ (1)

where $F^{D_{2}O}$ = 0.8607 is the molar fraction of solution D_2_O, *n* is the number of amino acids and *p* is the number of prolines in the peptide excluding the first two N-terminal residues, and $m_{H^{+}}$ and $m_{D^{+}}$ are proton and deuteron masses. HDX Workbench does not estimate the uncertainty of ${\%E}_{i,X}^{peptide}\left(t_{HDX}\right)$, as meaningful uncertainty arises mainly from Type B sources (e.g., peptide sequence-specific chromatographic background noise from co-eluting peptides) and not from the orders-of-magnitude more precise mass spectrometer. Meaningful uncertainty for $D_{X}^{peptide}\left(t_{HDX}\right)$ is estimated by *post hoc* computation of the associated sample standard deviation, $s_{k}$, which will include the variances computed from the three ${\%E}_{i,X}^{peptide}\left(t_{HDX}=0\;s\right)$. (For each peptide, the three ${\%E}_{i,X}^{peptide}\left(t_{HDX}=0\right)$ measurements sum to ≈ 0%.) Furthermore, for each dataset we can compute a pooled estimate of the variance [38]:

$s_{p}=\sqrt{\frac{\sum_{N}\left(n_{k}-1\right){s_{k}}^{2}}{\sum_{N}\left(n_{k}-1\right)}}$ (2)

where *k* specifies a peptide in the dataset list, $n_{k}$ is the number of measurements per $D_{X}^{peptide}\left(t_{HDX}\right)$, and N is the number of peptides in the datasets. For these studies $n_{k}$ = 3 for nearly all $D^{peptide}\left(t_{HDX}\right)$.

The working datasets comprise ≈ 17,750 measurements obtained during seven experiments, each comprising three runs of six exchange times [29]. The results are organized into 16 datasets (Table 3). Experiments 1, 3, 5, and 7 used solutions containing only one protein and yield HDX-MS data for the isolated FcγRIa and each isolated aIL8hFc glycoform. Experiments 2, 4, and 6 used solutions containing mixtures of FcγRIa and a selected aIL8hFc glycoform. These solutions yield fragments containing HDX-MS information on the binding interaction between FcγRIa and the selected aIL8hFc glycoform. HDX-MS data from each experiment are organized into 16 datasets, each comprising fragments from FcγRIa receptor, the light chain of aIL8hFc glycoform, and the heavy chain of aIL8hFc glycoform.

Table 3 reports the percentage of the protein sequence observed by peptides. Datasets for proteins of like sequence (e.g., apo- and holo-, or G0F and G2F, etc.) are mutually unbalanced; that is, some sequences reported in one dataset may not be reported in another. Imbalances may arise from electrospray efficiencies and noise; interference from the co-elution of peptides, resulting in poor signal to noise or poor definition of centroids; differences in disulfide reduction efficiency; and other instrument effects. From these unbalanced datasets the user may construct balanced datasets comprised of matching peptide sequences. Balanced datasets that encompass all states will comprise 35 light chain peptides, 63 heavy chain peptides, and 40 FcγRIa receptor peptides.

**Table 3:** Summary of the HDX-MS experiments and the list of database file names.

Dataset #	Exp #	State	# of Meas	# of Peptides	Coverage, %	Pooled Dev. (*s*_p_), Da	Filename (.CSV)
1	1	apo-G0F_LC_	660	37	95	0.068	Dataset#1**_**apo-G0F**_**light**_**chain
2	2	holo-${G0F}_{LC}^{Fc\gamma RIa}$	916	51	93	0.069	Dataset#2**_**holo-G0F**_**light**_**chain(FcgR1a)
3	3	apo-G2F_LC_	916	51	93	0.076	Dataset#3**_**apo-G2F**_**light**_**chain
4	4	holo-${G2F}_{LC}^{Fc\gamma RIa}$	980	55	93	0.079	Dataset#4**_**holo-G2F**_**light**_**chain(FcgR1a)
5	5	apo-SAF_LC_	933	52	96	0.086	Dataset#5**_**apo-SAF**_**light**_**chain
6	6	holo-${SAF}_{LC}^{Fc\gamma RIa}$	986	55	93	0.103	Dataset#6**_**holo-SAF**_**light**_**chain(FcgR1a)
7	1	apo-G0F_HC_	1367	76	84	0.078	Dataset#7**_**apo-G0F**_**heavy**_**chain
8	2	holo-${G0F}_{HC}^{Fc\gamma RIa}$	1494	83	84	0.067	Dataset#8**_**holo-G0F**_**heavy**_**chain(FcgR1a)
9	3	apo-G2F_HC_	1384	77	83	0.147	Dataset#9**_**apo-G2F**_**heavy**_**chain
10	4	holo-${G2F}_{HC}^{Fc\gamma RIa}$	1566	87	84	0.087	Dataset#10_holo-G2F**_**heavy**_**chain(FcgR1a)
11	5	apo-SAF_HC_	1494	83	84	0.085	Dataset#11**_**apo-SAF**_**heavy**_**chain
12	6	holo-${SAF}_{HC}^{Fc\gamma RIa}$	1548	86	85	0.120	Dataset#12**_**holo-SAF**_**heavy**_**chain(FcgR1a)
13	7	apo-FcγRIa	791	44	64	0.098	Dataset#13**_**apo-FcgR1a
14	2	holo-FcγRIa^G0F^	933	52	79	0.052	Dataset#14**_**holo-FcgR1a(G0F)
15	4	holo-FcγRIa^G2F^	881	49	77	0.057	Dataset#15**_**holo-FcgR1a(G2F)
16	6	holo-FcγRIa^SAF^	899	50	77	0.091	Dataset#16**_**holo-FcgR1a(SAF)

### 3.5 Organization of the Deposited HDX-MS Data

The data entries within each dataset file are organized in rows, where the columns specify the parameters of each measurement. Table 4 is the glossary defining these parameters.

**Table 4:** Glossary of column header terms in the HDX-MS data files.

**Column Header**	**Definition**
START	Index number of the first residue in the peptide with reference to the sequence of the subject protein.
END	Index number of the last residue in the peptide with reference to the sequence of the subject protein.
SEQUENCE	Sequence of amino acids comprising the peptide. Protein modifications are described in file: *Glossary of Protein Modifications.pdf*.
CHARGE	Positive charge (*z*) of observed peptide.
Mono-Mass	Calculated monoisotopic mass in Daltons (Da) of the peptide.
RT_Window	Retention times (time window) in minutes over which chromatographic elusion data for the peptide was measured by the mass spectrometer.
Time	Duration in seconds of incubation ($t_{HDX}$) of protein in a solution containing $F^{D_{2}O}\;$D_2_O.
Mass_Uptake	Computed mass in Daltons of deuterium exchanged into peptide during period $t_{HDX}$.

## 4 Impact

These data have value for the development and testing of HDX-MS analysis software designed to determine quantitatively the degree of similarity among a set of proteins that differ in their post translational modifications. The data can also guide the development of computational simulations of deuterium uptake kinetics and the development of automated data evaluation algorithms. The original use of these spreadsheets was for a determination of the effects of glycosylation upon IgG1 dynamics and the differential interaction of IgG1 glycoforms with the human FcγRIa receptor [39].
